# Case of Chronic Otitis Media with Intracranial Complication and Contralateral Extracranial Presentation

**DOI:** 10.1155/2016/7810857

**Published:** 2016-09-07

**Authors:** X. Y. Yeoh, P. S. Lim, K. C. Pua

**Affiliations:** Department of Otorhinolaryngology, Penang General Hospital, Jalan Residensi, 10990 Penang, Malaysia

## Abstract

Intracranial complications of chronic otitis media have been on the decline with advent of antibiotics. Septic thrombosis of the sigmoid sinus is rarer compared to commoner complications such as otogenic brain abscesses and meningitis. This patient presented with recurrent infection after left mastoidectomy secondary to cholesteatoma and a contralateral internal jugular vein thrombosis with parapharyngeal abscess, which was drained. He recovered well postoperatively with antibiotics.

## 1. Introduction

Otitis media is potentially serious due to its life-threatening complications. The complications arising from this condition can be further divided into intracranial and extracranial. These complications, from being common with high morbidity and mortality rates, have become rare now with arrival of the antibiotic era. A retrospective study by Lund talks of the mortality rate due to intracranial complications being at 36% between 1939 and 1949, 6% from 1950 to 1960, and 0% from 1961 to 1971, demonstrating the drastic change in the incidence [1].

## 2. Case Report

**Figure 1:**
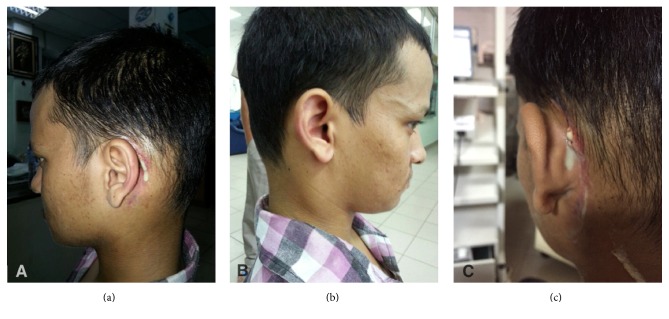
(a and c) Wound dehiscence of the left mastoidectomy site discharging pus. (b) Right neck fullness.

**Figure 2:**
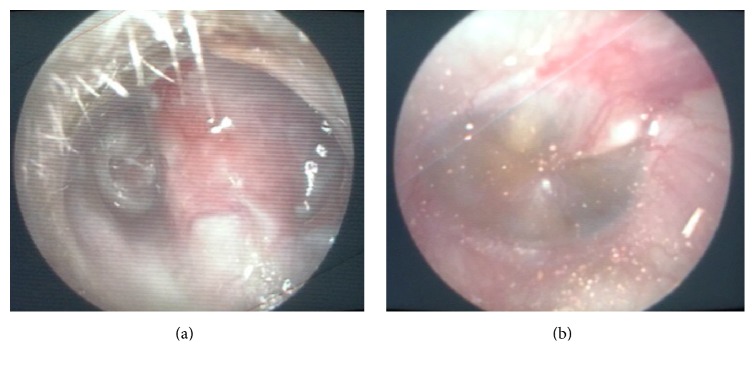
(a) Left otoscopy showing mucopus at the mastoid cavity and external auditory canal. (b) Right inflamed and dull tympanic membrane.

**Figure 3:**
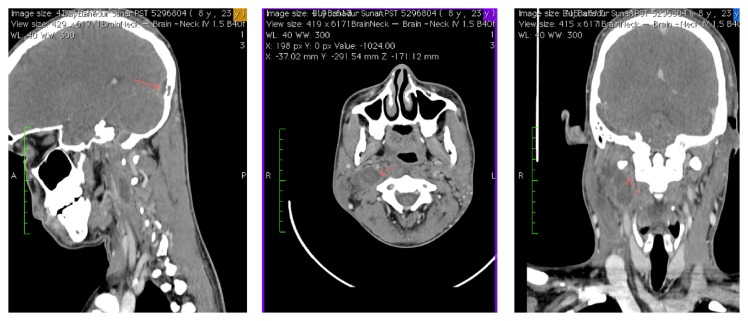
CECT showing right IJV thrombosis with right parapharyngeal abscess and delta sign at the transverse sinus.

A 23-year-old Nepali, with history of left modified radical mastoidectomy 2 months prior for cholesteatoma, presented with one-week history of fever, right otalgia, neck pain, and right neck swelling, with reduced neck movement. On examination, he appeared ill with high spiking fever; there was a presence of House Brackmann grade II facial nerve palsy on the left (present only postoperatively) and the left postauricular wound dehiscence was discharging pus. He also had torticollis to the right, associated with fullness over the right upper neck (Figure 1). Otoscopy on the left revealed copious amounts of mucopus in the middle ear and mastoid cavity and, on the right, an inflamed but dull tympanic membrane (Figure 2). Due to financial constraints, a myringotomy was performed on the right ear, yielding only mucoid material, and a failed aspiration over the fullness of the right neck was done. An exploration of the left wound was performed under local anaesthesia; draining pus and packing was done. His fever persisted, and an urgent brain and neck Contrast Enhanced Computed Tomography (CECT) was done, showing soft tissue within the left mastoid cavity, right parapharyngeal abscess, and bilateral internal jugular vein (IJV) thrombosis (Figure 3). Patient was subjected to drainage of the right parapharyngeal abscess under general anaesthesia. He became afebrile immediately postoperatively, and the torticollis resolved. Pus culture of the left postauricular wound grew* Pseudomonas aeruginosa* and of the right neck grew* Bacteroides* spp. He completed 10 days of IV Rocephine, Amikacin, and metronidazole and was discharged with oral antibiotics after secondary suturing was done over his right neck wound. He decided to continue his treatment in Nepal.

## 3. Discussion

Chronic suppurative otitis media (CSOM) affects 65–330 million individuals with draining ears, and accounts for 28000 deaths in 1990. The Western Pacific and Southeast Asian regions contribute 85–90% of this global burden from CSOM, with India and China accounting for most cases [2]. The majority of intracranial complications were caused by chronic otitis media and cholesteatoma (95.8%), and these complications occur more frequently in the first three decades of life with a higher incidence in males [3]. The commonest to occur are meningitis and brain abscess (temporal or cerebellar) and one or more complications may present in a single patient [4, 5]. They may present with headache, neck stiffness, vomiting, and fits associated with otorrhea and decreased hearing. However, these may be difficult to recognize and present atypically and more subtly as the symptoms can be masked by use of antibiotics. The commonest presentation of patients with lateral sinus thrombosis is sustained or spiking fever, associated with otorrhea, postauricular oedema, and otalgia [6], which were evident in this patient.

Sinus thrombosis occurs by bone erosion of the mastoid over the sinus, due to either cholesteatoma or granulomatous processes, forming a perisinus abscess. This abscess creates pressure on the bone, causing necrosis on the anterior portion of the sinus and the intima, with adherence of fibrin, red blood cells, and platelets, forming a mural thrombus. This thrombus might propagate towards the jugular vein bulb, and to other sites, or subcutaneous tissue, or it might throw emboli [7]. This patient had a left cholesteatoma with sigmoid sinus thrombosis, with retrograde propagation to the transverse sinus and contralateral sigmoid sinus, and IJV, resulting in parapharyngeal abscess formation over the right side. Lemierre's syndrome, according to a systematic review, is rarely due to middle ear or mastoid infections (2%), commoner causes being the tonsil, pharynx, or the chest. However, this patient's presentation of neck pain and swelling is the commonest for patients with IJV thrombosis [8].

Organisms involved in septic IJV are determined by the etiology. In intravenous catheter related IJV, the most likely organism is* Staphylococcus aureus*, and, in oropharyngeal infections, anaerobes are common. In otologic infections,* Proteus* and* Pseudomonas* are the most common organisms isolated [9]. This patient grew* Pseudomonas* from cultures of the left mastoidectomy site. CECT aids in the diagnosis of intracranial complications of otitis media, be it an abscess or a sinus thrombosis, which will demonstrate the “delta sign” (central nonenhancing clot surrounded by enhancing dural sinus wall) [10]. It is therefore imperative that CECT or an MRI be performed if sinus thrombosis is suspected as a plain HRCT of the temporal region, which is normally performed for patients with cholesteatoma undergoing surgery and would miss this fairly rare intracranial complication nowadays.

This patient's presentation was interesting as he presented with a unilateral discharging ear, he was diagnosed to have cholesteatoma, and mastoidectomy was performed after a HRCT temporal. He has currently presented with an extracranial extension of an intracranial complication on the contralateral side, and this septic thrombosis has caused the formation of a parapharyngeal abscess, which was adequately addressed with drainage.

In infected IJV thrombosis, the primary site of infection should be treated first; for example, neck abscesses should be drained and mastoidectomy should be done for mastoiditis [9], as in this patient. Most patients with infected IJV thrombosis do well on antibiotics alone, and the choice depends on the most likely organism. Migirov et al. have demonstrated that combination antibiotics are effective in treating intracranial complications in his series as many of his patients have been prescribed antibiotics prior to presentation [11]. In the case of a primary ear infection, the patient should be treated with Amikacin to cover Gram-negative organisms [9]. This patient was started on appropriate antibiotics, namely, Rocephin, Amikacin, and Metronidazole, and he responded to combined medical and surgical treatment that was rendered.

## 4. Conclusion

A contralateral presentation of the right parapharyngeal abscess due to septic IJV thrombosis resulting from chronic otitis media is rare. A high index of suspicion is required for diagnosis for proper treatment to be initiated.
